# Successful Prepectoral Breast Reconstruction in a Patient With Systemic Sclerosis

**DOI:** 10.7759/cureus.15996

**Published:** 2021-06-28

**Authors:** Shawhin R Shahriari, Amanda C Ederle, Cees T Whisonant, Gregory Borah, Jeffrey Wu

**Affiliations:** 1 Division of Plastic, Reconstructive, Hand and Burn Surgery, Department of Surgery, University of New Mexico Hospital, Albuquerque, USA; 2 Internal Medicine, University of Arkansas for Medical Sciences, Little Rock, USA; 3 Surgery, University of New Mexico School of Medicine, Albuquerque, USA; 4 Division of Plastic, Reconstructive, Hand, and Burns Surgery, Department of Surgery, University of New Mexico Hospital, Albuquerque, USA

**Keywords:** systemic sclerosis, prepectoral breast reconstruction, connective tissue disease, plastic surgery, breast reconstruction

## Abstract

Patients with connective tissue diseases have been shown to be at higher risk for complications after surgery. In this report, we describe a case of a patient with long-standing, stable systemic sclerosis (SS), diagnosed approximately 28 years ago, who underwent nipple-sparing mastectomy and immediate reconstruction with prepectoral tissue-expander placement. She subsequently had uneventful implant-based reconstruction with adjunctive fat grafting. To our knowledge, this is the first reported case of implant-based prepectoral reconstruction after mastectomy in a patient with SS.

## Introduction

Systemic sclerosis (SS) is a complex connective tissue disease (CTD) involving tissue fibrosis and vasculopathy in multiple organ systems [1].  Like other CTDs, SS is predominantly seen in women with a prevalence of 2.6 per 10,000 in the general population [2]. Clinical manifestations including fatigue, musculoskeletal inflammation, pulmonary fibrosis, skin tightness, itching, and gastroesophageal reflux disease (GERD) may be apparent at presentation [1].  In addition to the complex systemic disease, the medical management of this condition includes the use of disease-modifying anti-rheumatic drugs (DMARDs), which delay wound healing and cause iatrogenic immunosuppression, along with non-steroidal anti-inflammatory drugs and steroids [3-6]. SS patients are perceived to be higher-risk surgical candidates, especially when they are being actively treated with DMARDs or steroids.  

Concerns about impaired wound healing and increased infection risk due to immunosuppression, along with hypercoagulability, have led to the surgical community’s hesitance to perform elective breast reconstruction on these patients. Upon reviewing studies specifically in the setting of CTD and breast reconstruction, findings suggest that CTD is a risk factor for both increased complications overall and for prolonged hospital length of stay [7]. Breast reconstruction literature is consistent with the surgical literature in that these CTD patients are at overall higher risk for complications. 

We present a successful case of prepectoral implant-based reconstruction after mastectomy for cancer in a patient with SS.  In this patient, a right-sided nipple-sparing mastectomy was performed with immediate prepectoral breast tissue expander, followed by placement of a final prosthesis at a later time. Though the patient experienced minor wound-healing problems after the initial stage of the reconstruction, this case represents a successful breast reconstruction with prepectoral placement of a breast implant in a patient with SS. 

## Case presentation

A 63-year-old female with a past medical history of SS and a diagnosis of right breast cancer presented for nipple-sparing mastectomy and tissue expander insertion.  The patient’s SS clinical manifestations included Raynaud’s phenomenon, interstitial lung disease, and GERD and an associated gastrointestinal dysmotility disorder.  Further medical history included osteoporosis and well-controlled hypertension. 

The patient was diagnosed with right breast invasive lobular carcinoma arising in a small focus of pleomorphic lobular carcinoma in situ, estrogen receptor negative, progesterone receptor negative, and human epidermal growth factor receptor 2 of 2+. Staging of the cancer was determined to be T1B, N0, M0 clinically and she was referred to oncology for further management. 

At the time of her initial breast cancer diagnosis, she was not actively on any DMARDs or steroid medications. Previously she had been on courses of prednisone, methotrexate, and d-penicillamine to treat her symptoms, approximately 30 years prior to her breast cancer diagnosis. Her Raynaud’s digital symptoms were treated with nifedipine, along with conservative measures, such as hand warmers. Her GERD symptoms were well controlled on a proton-pump inhibitor as well. She had interstitial lung disease secondary to SS, but her diffusion capacity had not changed significantly over the previous four years, once she had established aggressive care treatment at our university. Pulmonary evaluation indicated that her pulmonary hypertension was unlikely to complicate anesthesia. Given the long course of her disease process, this indicated that her sclerotic disease was progressing slowly, which was seen as a good prognostic indicator. 

Due to the patient’s long-standing SS and interstitial lung disease, mastectomy was thought to be the best option. Lumpectomy and radiation therapy were not recommended due to the possibility of exacerbating wound-healing issues and worsening pulmonary function if she were to have chest wall radiation therapy. The patient subsequently underwent right breast nipple-sparing mastectomy, sentinel lymph node biopsy, and prepectoral insertion of a tissue expander with an acellular dermal matrix wrap. 

Two months after initial operation, she progressively developed a small incisional wound on her right breast, which subsequently expanded in size without infection. Upon discussion with the rheumatology, it was determined that this wound-healing problem was not consistent with a rheumatologic wound-healing problem. This wound was first managed with local topical care but ultimately required skin debridement and primary closure of the right breast wound. At the time of skin debridement, the implant pocket was examined and there was no gross evidence of infection, and cultures were negative. Her breast incision subsequently healed well without further complications.   

Nine months after initial mastectomy, tissue expander placement, and subsequent expansion, the tissue expander was exchanged for a permanent implant. At that time, right breast fat grafting was performed and a left breast mastopexy was undertaken for symmetry. This surgical intervention was uneventful and she healed in a normal manner on both breasts. 

**Figure 1:**
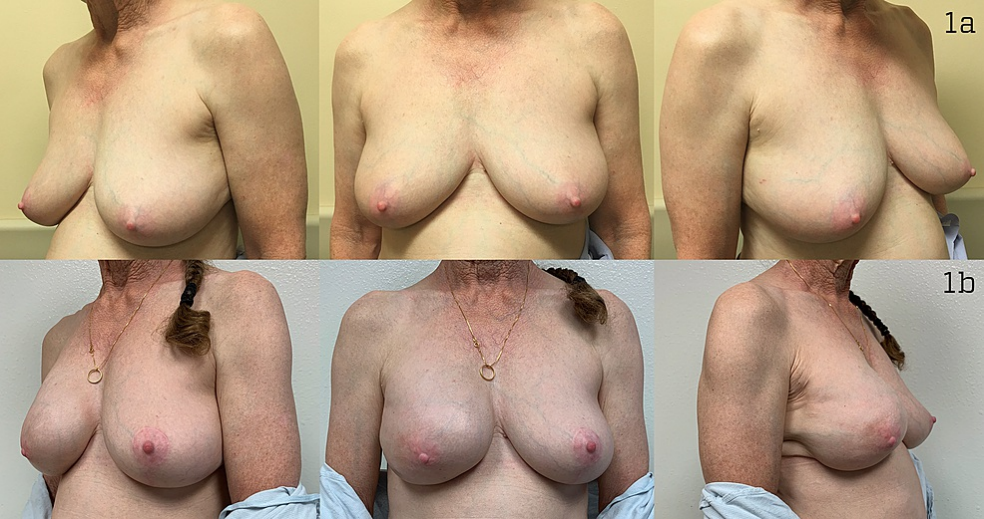
Preoperative and final reconstruction after right breast nipple-sparing mastectomy and prepectoral implant reconstruction, and left breast mastopexy for symmetry. 1a: Preoperative photos prior to nipple-sparing mastectomy and prepectoral implant-based reconstruction, oblique and anteroposterior views. 1b: Final reconstruction, 21 months after right breast nipple-sparing mastectomy, 11 months after right breast final prepectoral implant-based reconstruction and left-sided mastopexy, oblique and anteroposterior views.

Over a year after her final procedure, the patient continued to do well without any wound-healing problems (Figure 1). Of note, the patient had no complications postoperatively attributed to her underlying SS. She has not had any recurrence. She was ultimately satisfied with her final reconstructive outcome.

## Discussion

SS is an autoimmune disease that involves widespread fibrosis of tissues, including the blood vessels, kidneys, lungs, skin, gastrointestinal tract, nerves, and joints [1]. Clinical manifestations of the disease include arthropathies, rashes, ulcers, interstitial lung disease, pulmonary fibrosis, chronic obstructive pulmonary disease, and ischemic and immune-mediated changes to blood vessels [7]. These patients are often in hypercoagulable states, and have factors such as lupus anticoagulant, anticardiolipin, and antiphospholipid antibodies, all of which contribute to the hypercoagulability [1]. 

SS is more than a single disease; it is a spectrum of diseases with different manifestations and severities that ultimately drive treatment for these patients. The classic characteristics of SS are fibrosis and atrophy of the skin, and increased collagen deposition in internal organs, along with vasculopathy, although the clinical manifestation is variable. Classically, Raynaud’s phenomenon and GERD present early in the disease process. Fortunately, the more morbid manifestations of the disease (e.g. scleroderma renal crisis, pulmonary hypertension, and Raynaud's phenomenon) are treatable. Early diagnosis has been the focus of the rheumatology community, which allows for appropriate medical management earlier in the disease process. There are multiple subtypes within SS, each with their own specific constellation of symptoms. As a group the literature suggests that patients with SS in general have a higher mortality than other CTDs [1]. 

Concerns about skin and organ wound healing, infection risk, and clotting abnormalities have led to the surgical community’s hesitance to perform surgical procedures, including breast reconstruction, on these patients. For these reasons, there continues to be a paucity of studies exploring the optimum management, complications, and clinical success in patients with CTD. Little data exist specifically for patients with SS undergoing breast reconstruction following mastectomy.  

There are multiple explanations as to why patients with CTDs have increased complications, including wound-healing difficulties. The transforming growth factor beta (TGF-β) pathway is commonly affected in those with CTDs, and TGF-β is known to be a key molecule in the pathophysiology of wound healing [8]. Studies have shown that patients with CTD experience longer length of hospital stays, higher postoperative wound dehiscence, and higher rates of infection in the setting of immediate autologous breast reconstruction and also in the setting of immediate implant-based reconstruction [9]. The data did suggest that there was a higher complication rate with implant-based reconstruction in terms of wound dehiscence [9]. When looking at CTD overall, it has been shown to be a significant risk factor for wound dehiscence and increased length of stay [9]. 

Many of these CTDs, including SS, are clinically managed with immunosuppression.  Specifically, the use of DMARDs has become critically important to the treatment of these diseases.  DMARDs generally act by potent immunosuppression of T-cells or antimetabolite therapy. Thus, this raises the concern of increased risk of infection and delayed wound healing [3-6].  However, a recent study demonstrated that patients with CTDs on immunosuppressive therapies were at significantly lower risk of minor complications than untreated CTD patients, suggesting that the benefits of immunosuppressive therapy may outweigh the risks associated with CTDs [10]. 

Steroids are also often used in these patients, and steroids are a well-known inhibitor of wound healing. The use of vitamin A supplementation is an essential part of treating any patient on steroids who will be having surgery in order to counteract the negative effects on wound healing [9].

Of note, our patient was not actively on any immunosuppressive medications; her disease was stable and did not require acute DMARDs or steroids to treat it. Although SS and CTD patients are often labeled as at higher risk, ultimately there is a spectrum of diseases. Laboratory testing was negative in our patient; however, based on her clinical presentation, the patient met the criteria set forth in 2013 by the American College of Rheumatology/European League Against Rheumatism for classification of SS [8]. Since our patient did not have severe disease based off of her clinical evaluation by rheumatology (i.e. she had been diagnosed for more than 30 years and the disease was not progressing significantly), she was considered a good candidate for breast reconstruction. These subtleties do play a role in patient selection; if the SS was uncontrolled or she was requiring an extensive immunosuppressive regimen, we would have been much more hesitant in terms of offering prepectoral breast reconstruction. This also highlights the importance of working with our colleagues in rheumatology, making the care of these patients a multidisciplinary effort. 

Previous studies have not looked specifically at implant breast reconstruction in the setting of SS, and it is reasonable to say that articles looking at large databases of patients with CTD do not include prepectoral reconstruction, specifically due to the shift in the plastic surgery community away from prepectoral reconstruction as seen from 1985 to 2015; the studies reviewed related to CTD and breast reconstruction all had databases that fall within this subpectoral timeframe [11].  

When reviewing the published literature, it is not known whether there is a significantly increased risk of complications, specifically due to small sample size in studies looking at breast reconstruction in this patient population. Another limitation of previous studies has been that they focused on CTDs as a whole, rather than on the outcomes associated with specific disease processes, such as SS. Future studies can investigate operative outcomes of implant breast reconstruction in more patients with SS and other CTDs. 

## Conclusions

To our knowledge, this is the first case report of a successful prepectoral implant-based reconstruction after mastectomy in a patient with SS, or for any CTD. This case demonstrates that SS is not an absolute contraindication to prepectoral implant reconstruction with fat grafting after mastectomy. Breast reconstruction patients in fact should be evaluated on the spectrum of CTDs based on severity, rather than a binary scale of having a CTD or not.  Although there was a minor wound-healing difficulty after the tissue expander was placed, these were not believed to be due to the patient’s underlying SS. After minor wound surgery the patient ultimately tolerated the second-stage final reconstruction well. Fat grafting at the time of the second procedure was also uncomplicated and may have positively influenced the wound-healing outcome. 

Prepectoral breast implant placement in a two-stage reconstruction consisting of tissue expander placement followed by permanent prosthesis placement with fat grafting can be tolerated well and is certainly a viable option in patients with milder-severity SS.
